# Associations of working experience as public health physicians allocated in the rural area and mental health in young general practitioners

**DOI:** 10.1192/j.eurpsy.2025.880

**Published:** 2025-08-26

**Authors:** J.-Y. Yun

**Affiliations:** 1 Department of Psychiatry, Seoul National University Hospital; 2 Yeongeon Student Support Center, Seoul National University College of Medicine, Seoul, Korea, Republic Of

## Abstract

**Introduction:**

Young general practitioners allocated in the rural area sustain the community healthcare frontline and endure the limited medical resources and social supports. Physician mental health could affect the quality of healthcare.

**Objectives:**

We examined the association of work-related experience with mental health in young public health physicians in the rural area.

**Methods:**

Study promotional document was posted on the Korean Association of Public Health Doctors (PHD) website (http://kaphd.org) on July 2023. Three-hundred PHD (among the total 1,256 PHD) completed a web-based self-reported questionnaire on demographics, working experiences, and mental health on a first-come, first-served basis. Adjusted odds ratios(aOR) of depression, anxiety, lower self-esteem, and distress for working experiences in PHD were calculated in the multivariable logistic regression model with the best fit combinations of significant explanatory variables (P<0.05) in univariate logistic regression.

**Results:**

Distress, depression, anxiety, and lower self-esteem were reported by 39.7%, 25.3%, 11.3%, and 8.7% of the PHDs, respectively. Higher odds of worse mental health in PHD were associated with patient-physician conflicts at workplace, isolated residence from local facilities, and higher workload. Conversely, perceived expertise utilization and social connections with peers were related to the lower odds of worse mental health in PHD.

**Conclusions:**

This study highlights the needs of governmental policy targeting the young general practitioners in rural area to enhance the expertise utilization and social connectedness, and to reduce the patient-physician conflict.

**Disclosure of Interest:**

None Declared

